# Supplementary material for: Global alignment reference strategy for laser interference lithography pattern arrays
Source: Microsyst Nanoeng. 2025 Mar 4;11:41. doi: 10.1038/s41378-025-00889-4 (PMC11880522; doi:10.1038/s41378-025-00889-4)
Supplement: Supplementary file 1 — Supplementary Information [file 41378_2025_889_MOESM1_ESM.docx]

**Supplementary Information for**

**Global Alignment Reference Strategy for Laser Interference Lithography Pattern Arrays**

Xiang Gao^1^, Jingwen Li^1,2^, Zijian Zhong^1^, and Xinghui Li*^1,2^

^1^Shenzhen International Graduate School, Tsinghua University, University Town of Shenzhen, Nanshan District, Shenzhen, 518055, Guangdong, China

^2^Tsinghua-Berkeley Shenzhen Institute, Tsinghua University, University Town of Shenzhen, Nanshan District, Shenzhen, 518055, Guangdong, China

*Corresponding author: li.xinghui@sz.tsinghua.edu.cn

**1 Supplementary Equations**

The period of the reference fringes is

$$\begin{aligned} d_{F}=\frac{\lambda}{2\sin\frac{\varphi}{2}},\#\left( S1 \right) \end{aligned}$$

where $\lambda$ is the wavelength of the beam, $\varphi$ is the small angle introduced.

If the relative deflection of exposure beam and the substrate along the *y*-axis is $\Delta\theta_{y}$, the period $d_{g}$ of the exposure fringes changes to

$$\begin{aligned} d_{g}=\frac{\lambda}{cos\Delta\theta_{y}\left( 2\sin\omega\right)},\#\left( S2 \right) \end{aligned}$$

where $\omega$ is the angle between the beam and the substrate normal.

The tilt angle $\alpha_{F}$ of the reference fringes changes to

$$\begin{aligned} \alpha_{F}=\arctan\frac{\tan\left( \omega_{d1}-\omega_{d2} \right)}{\tan\varphi},\#\left( S3 \right) \end{aligned}$$

where $\omega_{d1}$ and $\omega_{d2}$ are the angles between the substrate normal and the $-1$st-order diffraction beam of beam $B_{1}$, and the $-2$nd-order diffraction beam of beam $B_{2}$. Here,

$$\begin{aligned} \omega_{d1}=\arcsin\left( \sin\left( \arcsin\frac{\lambda}{2d_{r}}-\Delta\theta_{y} \right)+\frac{\lambda}{d_{r}} \right),\#\left( S4 \right) \end{aligned}$$

$$\begin{aligned} \omega_{d2}=\arcsin\left( \sin\left( -\arcsin\frac{\lambda}{2d_{r}}-\Delta\theta_{y} \right)+\frac{2\lambda}{d_{r}} \right),\#\left( S5 \right) \end{aligned}$$

where $d_{r}$ is the period of the reference grating.

If the angle between the two exposure beams changes, for example, if beam $B_{2}$ is deflected by $\Delta\theta$ along the *y*-axis, the period $d_{g}$ of the exposure fringes changes to

$$\begin{aligned} d_{g}=\frac{\lambda}{\sin\omega+sin\left( \omega+\Delta\theta\right)}.\#\left( S6 \right) \end{aligned}$$

The tilt angle $\alpha_{F}$ of the reference fringes can also be represented as

$$\begin{aligned} \alpha_{F}=\arctan\frac{\tan\left( \omega_{d1}-\omega_{d2} \right)}{\tan\varphi},\#\left( S7 \right) \end{aligned}$$

but $\omega_{d1}$ and $\omega_{d2}$ are

$$\begin{aligned} \omega_{d1}=\arcsin\frac{3\lambda}{{2d}_{r}},\#\left( S8 \right) \end{aligned}$$

$$\begin{aligned} \omega_{d2}=\arcsin\left( \sin\left( -\arcsin\frac{\lambda}{2d_{r}}-\Delta\theta\right)+\frac{2\lambda}{d_{r}} \right).\#\left( S9 \right) \end{aligned}$$

If the substrate rotates relative to the beam along the *z*-axis for an angle ${\Delta\theta}_{z}$, the tilt angle $\alpha_{g}$ of the exposure fringes changes to

$$\begin{aligned} \alpha_{g}={\Delta\theta}_{z}.\#\left( S10 \right) \end{aligned}$$

The period $d_{F}$ of the reference fringes changes to

$$\begin{aligned} d_{F}=\frac{\lambda}{\sin\varphi+\sin\left( \arctan\left( \frac{\lambda}{2d_{r}}\tan\left( 2\Delta\theta_{z} \right) \right) \right)}.\#\left( S11 \right) \end{aligned}$$

If there is a relative rotation between the two exposure beams, for example, if beam $B_{2}$ rotates by $\Delta\beta$, the tilt angle $\alpha_{g}$ of the exposure fringes changes to

$$\begin{aligned} \alpha_{g}=\arctan\frac{\tan\left( \varphi+\Delta\beta\right)}{2\sin\omega}.\#\left( S12 \right) \end{aligned}$$

The period $d_{F}$ of the reference fringes changes to

$$\begin{aligned} d_{F}=\frac{\lambda}{2\sin\frac{\varphi+\Delta\beta}{2}}.\#\left( S13 \right) \end{aligned}$$

**2 Supplementary Figures**

**
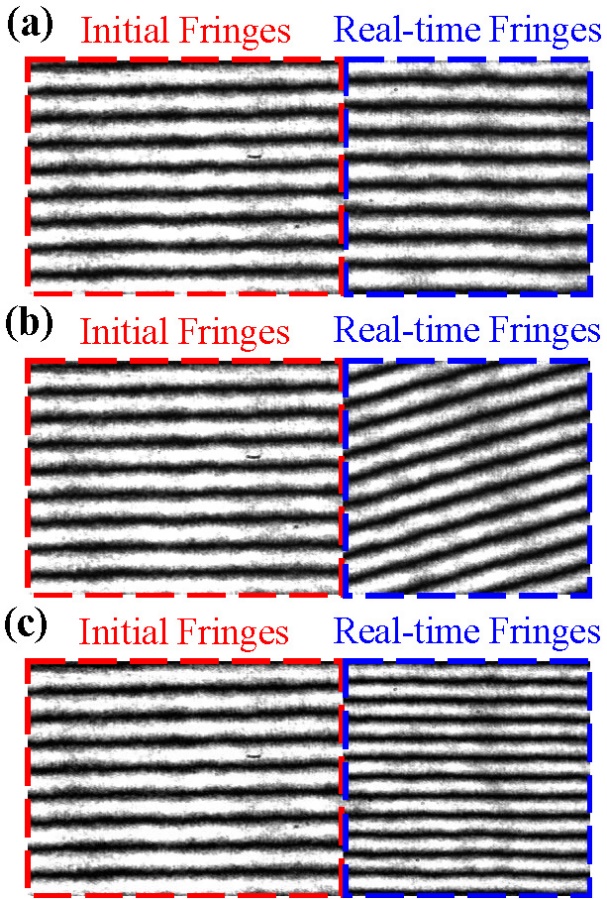
**

Supplementary Figure 1: Pictures of reference fringes: (a) When a phase error is introduced, the phase of the reference fringes will change; (b) When a period error is introduced, the tilt angle of the reference fringes will change; (c) When a tilt error is introduced, the period of the reference fringes will change.


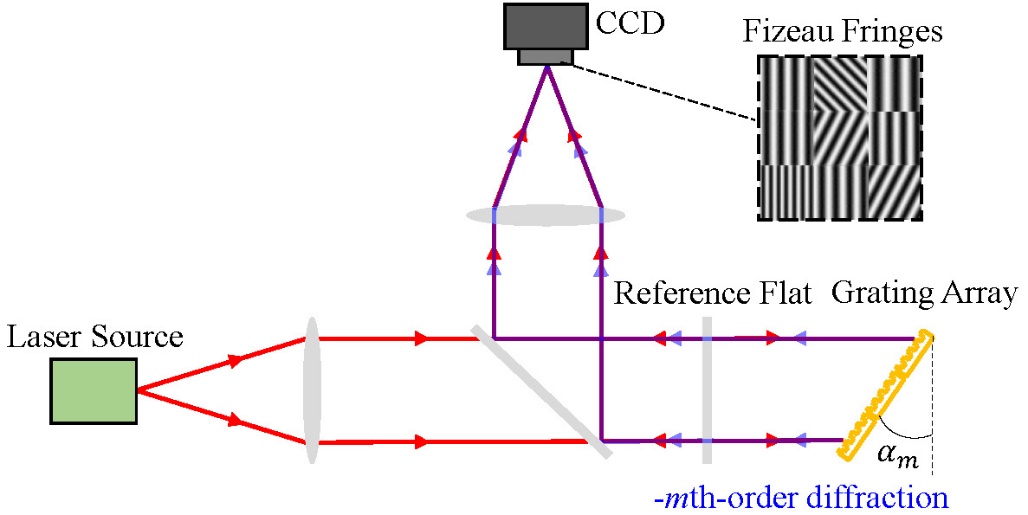


Supplementary Figure 2: Principle of measuring diffraction wavefronts of grating arrays using a Fizeau interferometer.


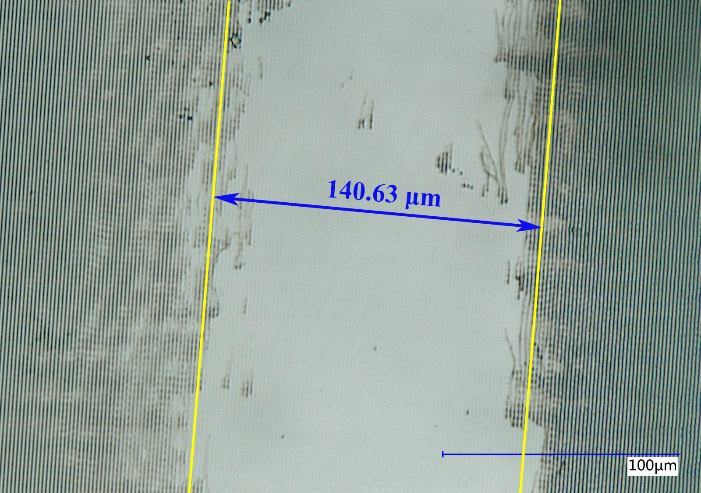

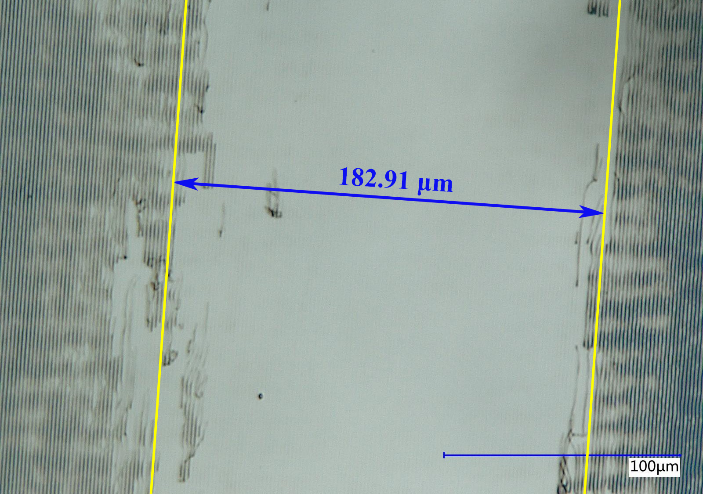


(a) (b)


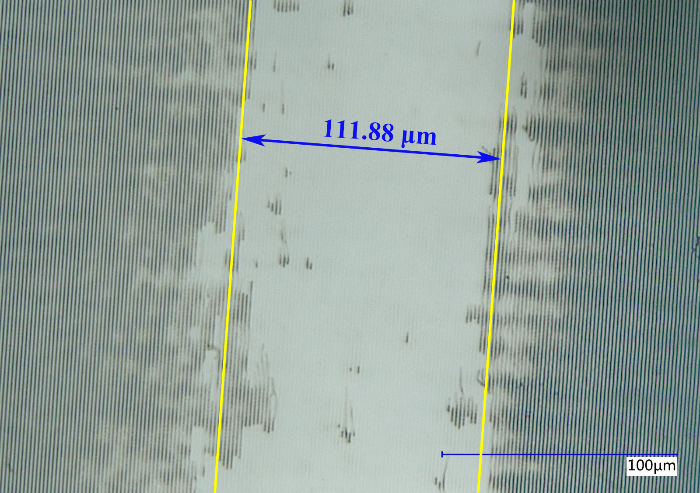

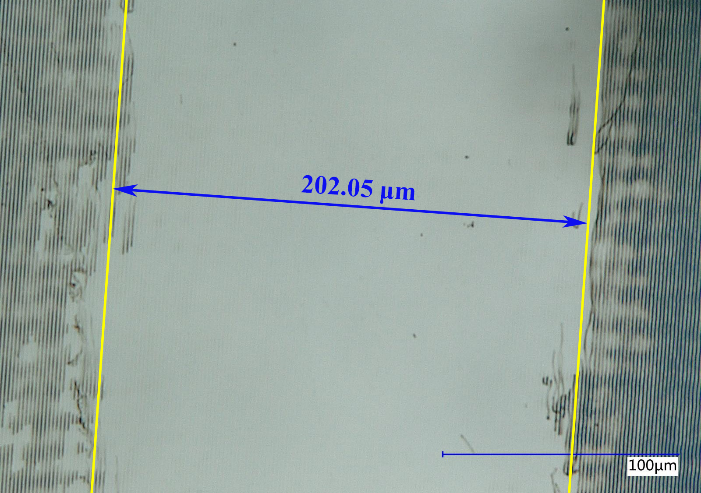


(c) (d)


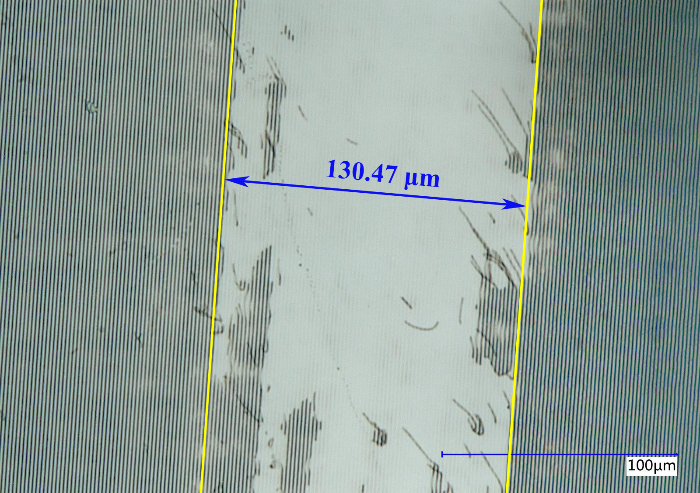

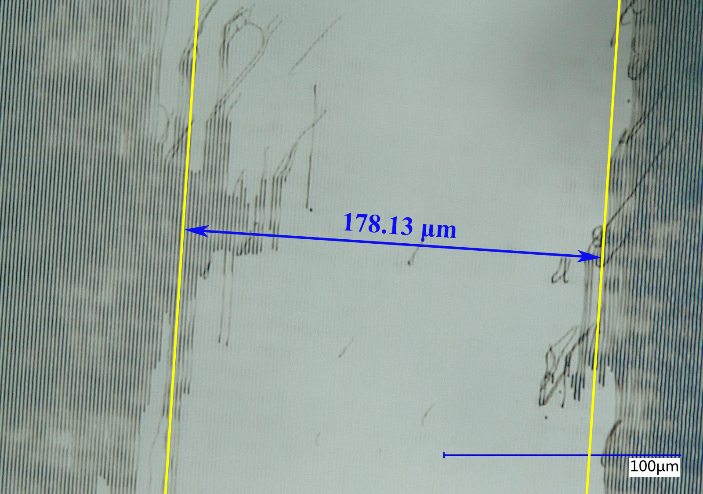


(e) (f)

Supplementary Figure 3: Transverse seams: (a) Between Regions 1 and 2; (b) Between Regions 2 and 3; (c) Between Regions 4 and 5; (d) Between Regions 5 and 6 (e) Between Regions 7 and 8; (f) Between Regions 8 and 9.


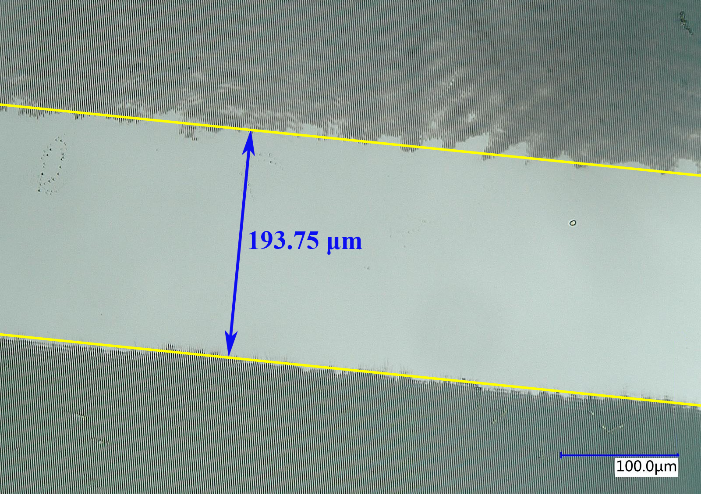

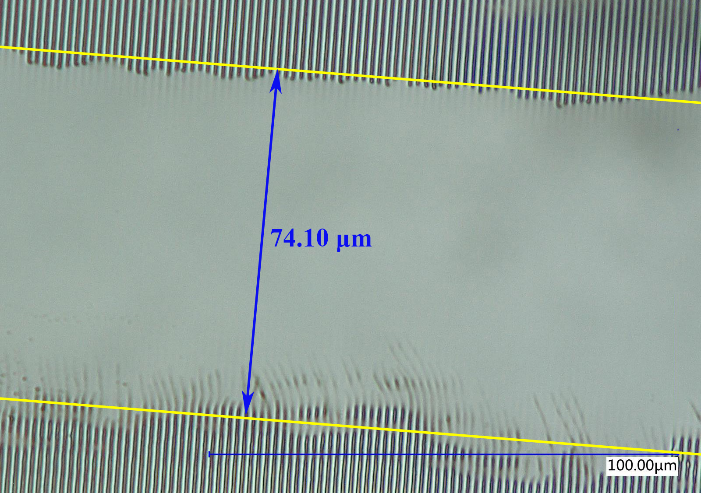


(a) (b)


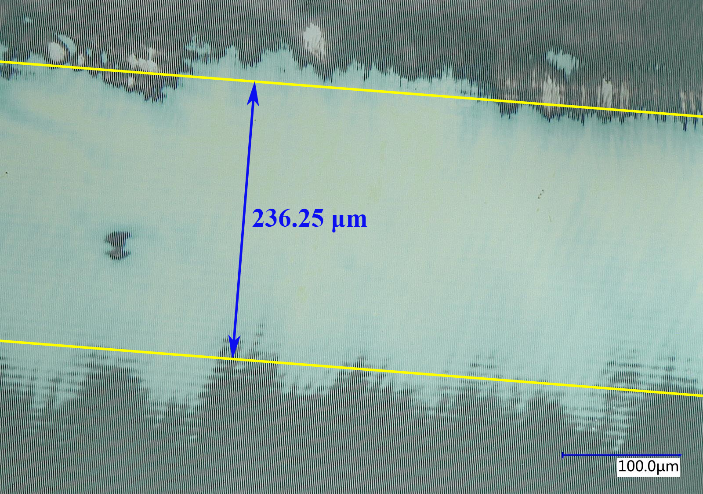

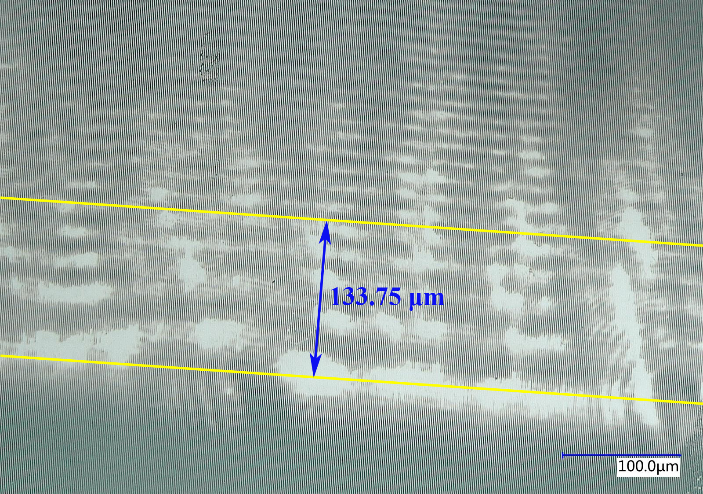


(c) (d)


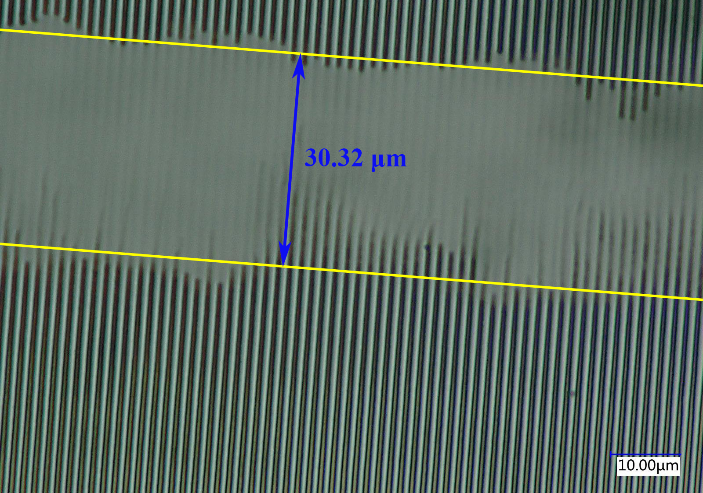

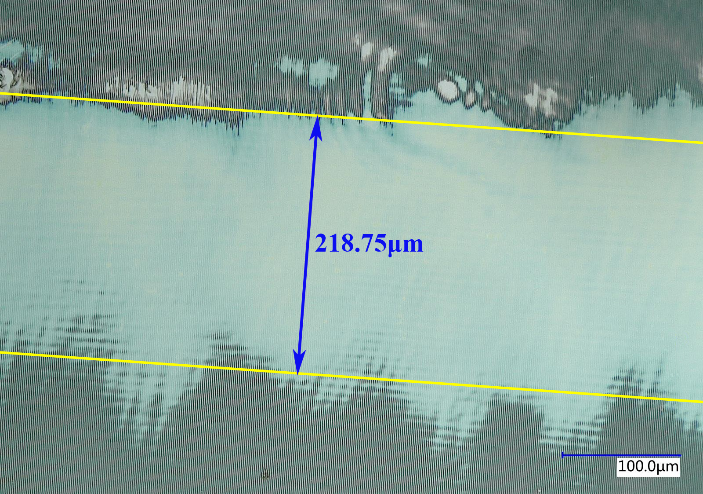


(e) (f)

Supplementary Figure 4: Longitudinal seams: (a) Between Regions 1 and 4; (b) Between Regions 2 and 5; (c) Between Regions 3 and 6; (d) Between Regions 4 and 7 (e) Between Regions 5 and 8; (f) Between Regions 6 and 9.
